# Guide for the application of the data augmentation approach on sets of texts in Spanish for sentiment and emotion analysis

**DOI:** 10.1371/journal.pone.0310707

**Published:** 2024-09-26

**Authors:** Rodrigo Gutiérrez Benítez, Alejandra Segura Navarrete, Christian Vidal-Castro, Claudia Martínez-Araneda

**Affiliations:** 1 Information Systems Department, Universidad del Bio-Bío, Concepción, Bio-Bío, Chile; 2 Computer Science Department, Universidad Católica de la Santísima Concepción, Concepción, Bio-Bío, Chile; University of the Chinese Academy of Sciences, CHINA

## Abstract

Over the last ten years, social media has become a crucial data source for businesses and researchers, providing a space where people can express their opinions and emotions. To analyze this data and classify emotions and their polarity in texts, natural language processing (NLP) techniques such as emotion analysis (EA) and sentiment analysis (SA) are employed. However, the effectiveness of these tasks using machine learning (ML) and deep learning (DL) methods depends on large labeled datasets, which are scarce in languages like Spanish. To address this challenge, researchers use data augmentation (DA) techniques to artificially expand small datasets. This study aims to investigate whether DA techniques can improve classification results using ML and DL algorithms for sentiment and emotion analysis of Spanish texts. Various text manipulation techniques were applied, including transformations, paraphrasing (back-translation), and text generation using generative adversarial networks, to small datasets such as song lyrics, social media comments, headlines from national newspapers in Chile, and survey responses from higher education students. The findings show that the Convolutional Neural Network (CNN) classifier achieved the most significant improvement, with an 18% increase using the Generative Adversarial Networks for Sentiment Text (SentiGan) on the Aggressiveness (Seriousness) dataset. Additionally, the same classifier model showed an 11% improvement using the Easy Data Augmentation (EDA) on the Gender-Based Violence dataset. The performance of the Bidirectional Encoder Representations from Transformers (BETO) also improved by 10% on the back-translation augmented version of the October 18 dataset, and by 4% on the EDA augmented version of the Teaching survey dataset. These results suggest that data augmentation techniques enhance performance by transforming text and adapting it to the specific characteristics of the dataset. Through experimentation with various augmentation techniques, this research provides valuable insights into the analysis of subjectivity in Spanish texts and offers guidance for selecting algorithms and techniques based on dataset features.

## Introduction

The explosive increase in the use of social media as a means of mass communication in the last decade [1, 2], has opened new research avenues for Natural Language Processing (NLP). Among these are the classification of texts with emotional intent (EI) and the identification of text polarity (SA). The approaches used in these NLP tasks include those based on Machine Learning (ML) such as Naïve Bayes (NB), K-Nearest Neighbor (KNN), and Support Vector Machine (SVM) [10] and the evolution towards those based on Deep Learning (DL) [3], which are data-driven approaches with high computational complexity [3]. However, to achieve their purpose, these models require substantial amounts of labeled data [4, 5], which poses a problem, as the task of manually labeling texts is time-consuming and resource-intensive [1, 6–8]. Therefore, alternatives are sought that allow for the acquisition of labeled data quickly, efficiently, and, as much as possible, without the need for human intervention. In this sense, data augmentation techniques, which were initially used in image analysis, are now used in text analysis to increase the sets of labeled data and thus improve the performance of classification models [9, 10].

This work is framed within the analysis of text subjectivity in Spanish, to analyze the effect on the performance of Machine Learning (ML) and Deep Learning (DL) classification models when data augmentation techniques are used. To achieve this objective, after reviewing the state of the art of text augmentation, the most used data augmentation techniques for texts will be applied to different datasets created by the SoMos (SOftware-MOdelling-Science) research group of the Universidad del Bío-Bío to evaluate the impact of augmentation on the classification of sentiments and emotions in the Spanish language with the most common ML and DL algorithms such as Support Vector Machine (SVM), Convolutional Neural Network (CNN), Long Short-Term Memory (LSTM), Bidirectional Long Short-Term Memory (BiLSTM), and Bidirectional Encoder Representations from Transformers pre-trained on Spanish Corpus (BETO). With these results, a guide is proposed for the selection of text augmentation techniques for sentiment analysis and emotion analysis tasks. The hypothesis that guides our work is described below “It is possible to improve the performance of classifiers based on Machine and Deep learning models for sentiment and emotions analysis in Spanish texts by employing data augmentation techniques.

The remainder of this work is structured as follows: The Related Works section provides a review of the literature on various taxonomies and data augmentation techniques. The Method section outlines the working hypotheses and a methodological approach for validation. The Experiments section details the phase of description and preparation of the datasets for continuing with the experimentation, considering the results derived from a baseline involving non-augmented data versus data augmented with the selected techniques. The Results section presents the overall results of the experimentation with the various data augmentation techniques on the datasets, also proposing a guide for selecting an augmentation technique and classification model based on the characteristics of a dataset. The final sections correspond to the discussion of the results, conclusions and future directions.

## Related works

**Fig 1:**
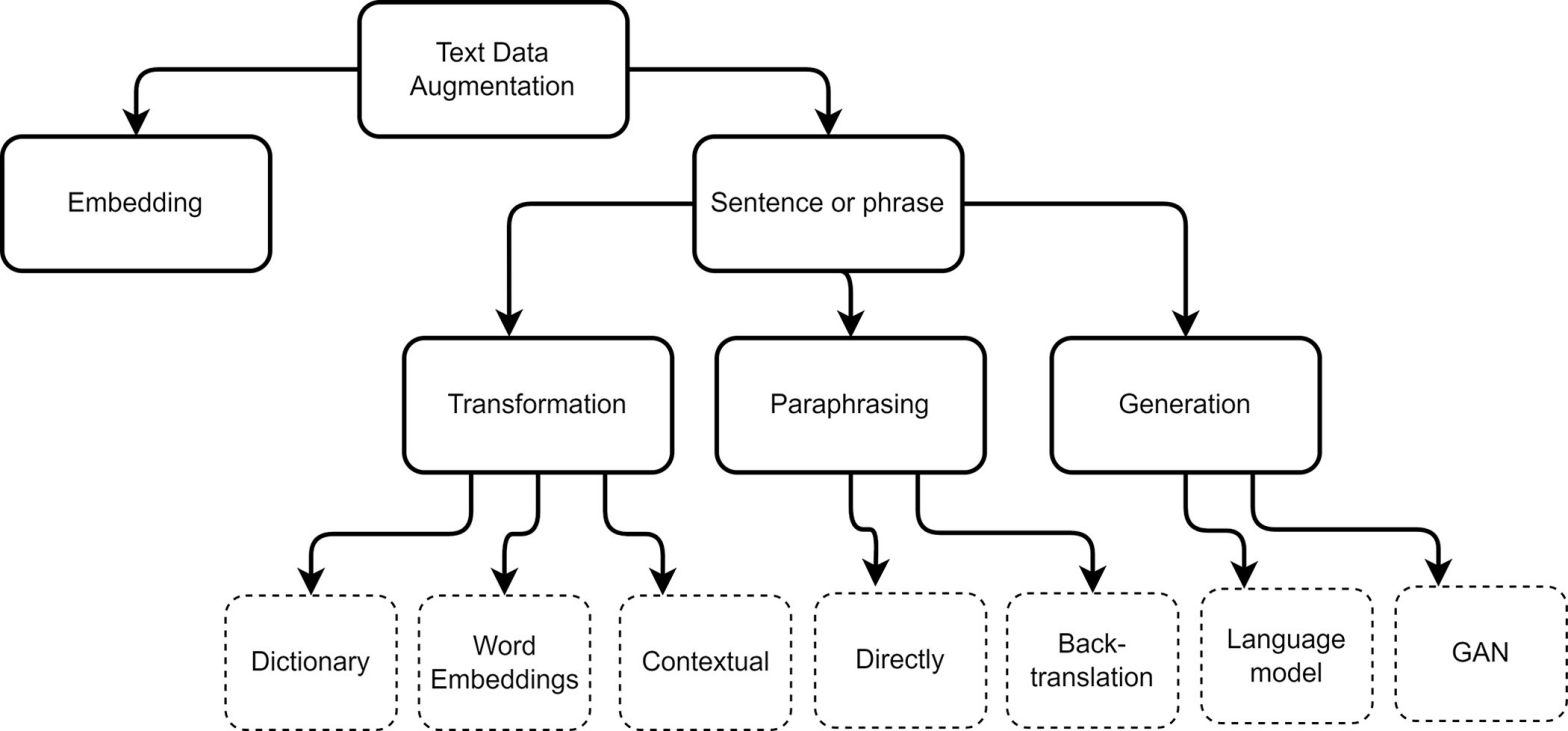
The taxonomy of data augmentation. Based on Queiroz *et al*. [17].

Data augmentation (DA) is a set of methods used to generate new data from a set of labeled data [11–14]. Initially, it was used to augment data in image processing [15], but over time, data augmentation techniques for text have been developed to improve the classification performance of ML and DL models. Text augmentation is used to address the issues of scarcity of labeled data and imbalance in the classes of a dataset [2], by generating new sentences from the existing ones in the dataset. This section will delve into the various text data augmentation techniques. Two taxonomies were reviewed that allow categorizing them. The first taxonomy, presented by Bayer *et al*. [16], defines two major categories: the *Data Space*, which groups techniques that perform augmentation directly on the data at the level of characters, words, phrases, and documents; and the *Feature Space*, usually represented through embeddings, which groups the techniques that perform augmentation through the manipulation of the vector representation of the data. This work focuses on augmentation methods based on Data Space. The second taxonomy reviewed is the one presented in the work of Queiroz *et al*. [17]. In it, a more comprehensive classification of augmentation techniques is shown by classifying them according to the type of augmentation performed on the dataset. Fig 1 offers a detailed view of this taxonomy. Under this last taxonomy, our work also shows the application of transformation, paraphrasing, and generation techniques.

### Data augmentation through sentence transformation

This variant mainly relies on operations of replacement, insertion, swapping, and deletion of words at random [18]. In the case of word replacement and insertion, the choice of these can be performed using dictionaries such as WordNet, word embeddings (WE) with Word2Vec [11], and the identification of the importance of words in a dataset using techniques like TF-IDF to preserve the original sentence. In Table 1, examples of augmentation through replacement, insertion, swapping, and deletion of words within a sentence are shown.

**Table 1:** Examples of sentence transformation, Balakrishnan *et al*. [11].

Original text	The quick brown fox jumps over the lazy dog	
Operation	Description	Example
**Random swapping (DRAWS)**	Two random words are selected and swapped.	The *lazy* quick fox jumps over the dog *brown*.
**Random deletion**	A random word is removed from the sentence.	The quick brown jumps over the lazy dog.
**Random insertion**	A new word is randomly introduced into the sentence.	The quick *sluggish* brown fox jumps over the lazy dog.
**Synonym replacement (PWSS)**	Select n words in the sentence and replace them with their synonym.	The quick sluggish *umber* fox jumps over the lazy dog.

In this work, a dataset is augmented using EDA (Easy Data Augmentation) [18], followed by a comparison between ML and DL classifiers. The reported results show that the DL algorithms used (CNN, RNN, BiLSTM, and BERT variants) perform better than ML algorithms (LR, NB, DT, RF, SVM) for both the original and augmented datasets. The performance of the DL algorithms with augmented data was 96% *accuracy* and 91.1% *F1-score*.

Furthermore, Chen e*t al*. [9] propose a variation of EDA that considers the information contained in emojis in tweets to preserve their semantics, and then perform SA using BiLSTM. The results show that the performance of the original dataset with the classifier is poor due to the low amount of labeled data it has; they show that with augmentation using EDA and emojis, their results improved significantly. Their *accuracy* and *F1-score* metrics are around 72%. Yuan *et al*. [19] propose a new adaptation of EDA, used to augment a dataset with 4 affective categories (*joy*, *anger*, *bored*, *sad*), to address the problem of semantic loss, the authors propose the use of TF-IDF to find the most important words in context. Then, they subject the augmented dataset to CNN classifiers, the results of their experimentation show that compared to their baselines, the proposed model improves the *accuracy* metric by 3%. Another work that focuses on the use of EDA is Lee *et al*. [20], in this work, they use *Knowledge Graphs* for the representation of sentences and the modification of them is based on the techniques used by EDA.

Dhiman *et al*. [12] use MOD-EDA to augment a dataset of tweets concerning the feelings of users regarding India’s public policies and the influence they have on election periods, then using BERT they perform the classification of the polarity of the tweets. To measure the performance of their model, they make combinations between the classifier with and without augmentation, obtaining the best results with MOD-EDA + BERT with percentages of 70% *accuracy* and 71% *F1-score*.

To improve classification results using BERT in conjunction with the English lexicon, Tahayna *et al*. [1] perform the replacement of idioms in English according to their meaning, thus obtaining new sentences. Using the *F1-score* as a metric, they report that their model proved an enhancement in ranking by over 10% compared to the baseline (76.98%) across a dataset of 150 tweets. Meanwhile, Li *et al*. [10] propose their algorithms for synonym replacement augmentation (PWSS) and word order exchange within a sentence (DRAWS), thus augmenting four public datasets concerning the SA task, as classifiers for their work using LSTM. The reported results show that the increase through DRAWS (11.49% Macro-F1) has better performance than PWSS (2.9% Macro-F1) with the dataset used. To perform SA on tweets in the Turkish language, Shehu *et al*. [17, 21] proposes three methods of augmentation, *shift*, *shuffle*, and a combination of both, and then subjecting the augmented dataset to ML and DL classifiers. Unfortunately, the authors do not delve into augmentation techniques, because their focus is their proposed classification model (HAN). Their results show that the use of DL algorithms obtains better classification results than ML algorithms. However, they show that, in terms of execution times and training, ML behaves better.

Liu *et al*. [22] use BiLSTM as a sentiment classification model in emails and the random replacement of words by combining WordNet and K-nearest Neighbor (KNN) to perform data augmentation of the dataset to balance polarity classes. Something interesting to highlight is that by replacing 20% of the words in a sentence and applying Linear Discriminant Analysis (LDA) and TF-IDF [14, 23] to maintain semantics, better augmentation results are obtained. The reported results show that with its augmentation model and the use of BiLSTM as a classifier, it improves *accuracy* between 1.5% and 10%. One of the studies that did not obtain significant improvements with the augmentation of the dataset for the classification of aspect categories is that of Almasre [24] with results of *F1-score* 66.3% for baseline and 66.1% on the same metric for the augmented dataset. The augmentation method used is based on replacing no more than 25% of random words in a sentence and employing cosine similarity to find the words to be used for replacement. As a classifier, they use a variant of BERT for the Arabic language. One of the problems they faced in their model was the considerable number of aspect categories that needed to be augmented (34) and the imbalance in 11 of them.

Another example of the application of data augmentation to solve the imbalance problem of a dataset is the one proposed by Ha *et al*. [25], their augmentation model uses synonym replacement based on the *Paraphrase Database* to then use CNN to detect three categories (*support*, *other*, *and oppose*) in a dataset relating to opinions made by U.S. citizens concerning to a clean power plan. After augmentation, their dataset was balanced at 2800 comments per category. The results were *accuracy* and *F1-score* 84% and 71%, respectively. Continuing with the use of augmentation for dataset balancing, Lee *et al*. [20] compares two augmentation techniques, EDA and Unsupervised Data Augmentation (UDA), to balance samples in *Knowledge Graphs* with polarity charge. The experimentation with EDA in the negative category showed minimal impact on ranking performance when compared to the outcomes achieved with UDA in the positive category for the identical dataset.

To perform the classification of SA in Chinese texts, Wang *et al*. [26] augment a dataset by replacing synonyms extracted from a thesaurus that they build for their model, showing that the problem of replacing synonyms with a low level of similarity is corrected and that it influences the classification task. Once the dataset is augmented, they perform SA using a hybrid model between CharCNN (which extracts the features) and SVM (which performs the classification). The results reported in their work show that with their augmentation and classification model, they obtain an *accuracy of* 95%.

To perform sentiment analysis on tweets that have idioms, Tahayna *et al*. [27] use SliDE (IBM lexicon) and a BERT classifier. In their work, they propose a method of increasing tweets by replacing idioms with their meaning, with which they report a ranking performance of 92% and a 16% reduction in classification error from the dataset without augmentation.

By using text augmentation by transformation techniques, Qudar *et al*. [6] replace 5% of words with synonyms, remove 10% of words, insert 5%, and swap 5% of the words in a sentence. To then apply a semi-supervised student-teacher model with which they carry out sentiment analysis. The results obtained by their model are 87.3% *F1-score* for the dataset *SemEval Aspect Sentiment Analysis* and 88.35% *F1-score* for a dataset of *Twitter*.

To improve the detection of sarcasm in short Arabic texts, Al-Jamal *et al*. [28] use two techniques for in-text data augmentation, *Random Swap*, and *Random Deletion* to balance the classes of your iSarcasmEval dataset [29]. To then detect sarcasm using BERT. However, their results were less than 60% on this task for the metric *F1-score*. They show that more robust labeled datasets need to be built to improve the detection of sarcastic comments.

A different approach to augmentation by transformation is introduced by Kraus *et al*. [30], where they perform *Random Swap and Random Insertion* dataset represented as a *Rhetorical Structure Tree*. For sentiment analysis, they use a DL classifier called *Discourse-LSTM* with which they get a performance improvement with the Rotten Tomatoes dataset of 4.27% in *F1-score*.

Kelsingazin *et al*. [31] propose two implementations of algorithms for the augmentation of data in text based on *Random Insertion and Random Deletion*. However, they do not provide details of the implementation of these. As for the classifiers used, they show that they use SVM and LR, but do not provide details of the implementation or the parameters used. Regarding the results, they show an improvement in ranking performance of 1% in the *F1-score*.

Iosifidis *et al*. [2] propose two augmentation techniques. The first of these performs word replacement in a statement by selecting the most similar ones according to the cosine similarity calculated from WE. The second removes words from the sentence except those that have sentimental weight and those that correspond to negation, to preserve the class of the original sentence. The authors propose the use of these techniques to increase the training time of their model to correct the imbalance of the classes at the time of training. Throughout their experimentation, they show that the replacement method based on WE do not behave as well as the method of word elimination, in this situation, they suspect that at the time of making the replacements the classes of some of the samples are changed (i.e., the meaning of the sentence is changed).

Duwairi *et al*. [13] report the most significant enhancement in performance, with a 42% improvement in *accuracy* after augmenting a dataset with product reviews in Arabic. The proposed augmentation method is based on the replacement of synonyms from Arabic-Wordnet and the application of syntactic rules specific to the Arabic language to generate new statements. In their experimentation, they evaluate the augmented datasets on three ML classifiers (NB, KNN, SVM) and increase the dataset up to ten times, achieving an increase in classification performance of more than 40% compared to the unaugmented dataset.

To keep the semantics of augmented statements, Feng *et al*. [32] propose two algorithms, the first of which performs probabilistic synonym replacement by selecting words from a lexicon. The second extracts the weight of the words in the context by using TF-IDF and replaces the one with the least weight concerning the context. For the classification, they use a CNN-based model, with which they achieve a 5% improvement in the classification concerning the baselines defined in the metric *accuracy*.

Santoso *et al*. [14] propose an extension of EDA with which they achieve an improvement of between 0.6% and 3.4% in *accuracy* concerning an unaugmented dataset. The improvement to EDA consists of the proposal of two algorithms for augmentation, the first by substitution while keeping the semantic information of the statement and the second by performing disambiguation using the *Adapted Lesk* algorithm. In their experimentation, they had problems with the neutral class because their model could not find the most important words in sentences with that class.

The work presented by Haralabopoulus *et al*. [7] encompasses the classification of emotions and polarity by LSTM and augmentation by antonym replacement, negation insertion, and permutation. Their augmentation model, unlike the others, tries to augment the dataset by changing the class in the augmented statement from the original statement. With this approach, they achieve a 4.1% performance improvement in *accuracy* over baselines. One of the findings in this work is that they do not bother to keep the semantics of the sentences, which goes in a completely different direction from the other articles.

Wei *et al*. [33] augment the dataset using synonym replacement, randomization, randomization, and random switching techniques, and then train a learning transfer model with two BERT instances, one as a student and one as a teacher. The results of their model show that they keep BERT performance *accuracy* for the SST2, YELP, and Amazon datasets. Recent editions of the Iberian Languages Evaluation Forum (IberLEF) have highlighted studies introducing data augmentation strategies for Spanish datasets using either BERT models [34] and Large Language Models (LLMs) [35]. These studies have shown promising results, in the first case using augmentation technique via Bayesian optimization (BO-TextAutoAugment) [36] and in the second using back-translation paraphrasing.

Despite the availability of Spanish and multilingual datasets involving sentiment analysis and emotion detection like those mentioned in Navas-Loro and Rodríguez-Doncel [37], there are a limited number of studies [5, 23, 34, 35] where data augmentation techniques are applied directly to the data set whose source language is Spanish without translation, as observed through the Related works section. Another challenge is related to the techniques used for augmentation and how they handle the preservation of the semantics of the original sample. To solve this problem, the use of the Term Frequency-Inverse Document Frequency (TF-IDF), Probabilistic Latent Semantic Analysis (pLSA), and word embeddings (WE), among others, is proposed in [38, 39].

### Data augmentation through paraphrasing

**Fig 2:**
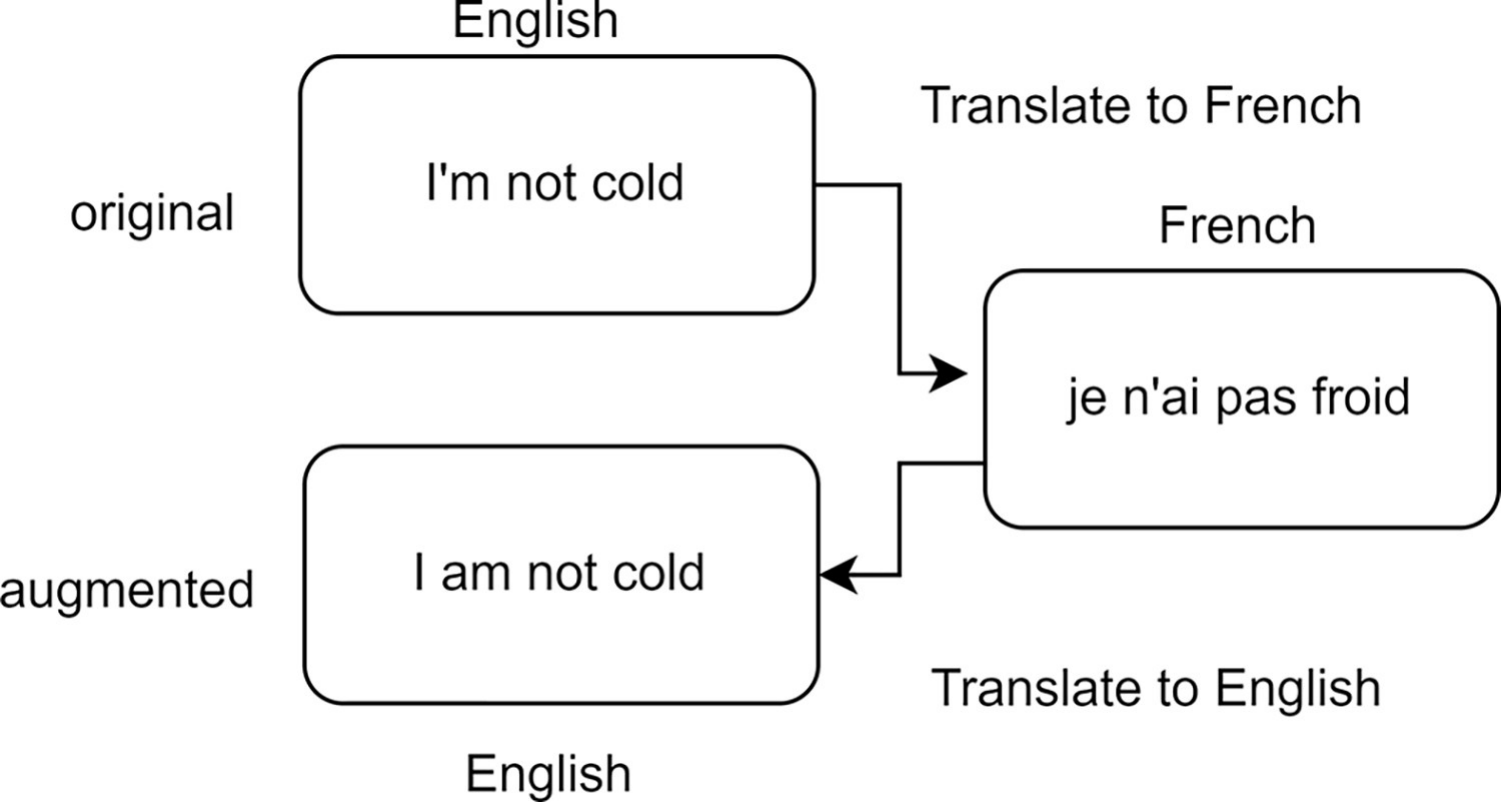
Example of back-translation based on Jacob and Shushma [8].

This strategy uses techniques such as back-translation (BT), which consists of translating sentences into one or more intermediate languages and then translating them back into the original language, as can be seen in Fig 2. By performing this operation, sentences are obtained with slight modifications produced by the effect of translation, but which keep the semantics of the original sentence. However, the quality of sentences generated using this method depends directly on the translation tools used [8, 17, 40, 41].

Among the works related to paraphrasing is the work of Krishnan *et al*. [41] which performs augmentation by translating sentences from English to Hindi, to increase the number of sentences that are used for its classification model, which consists of a teacher-student model using mBERT and XLM-R. The authors do not delve into the augmentation technique, only indicating that any translation or transliteration tool can be used. The results of their model in the test dataset manage to preserve or improve their performance concerning the baseline, obtaining for mBERT 61.35% and 66.24% for the Hindi and Malay languages in the *F1-score* metric and 62.23% and 76.46% for the same languages using XLM-R.

On the other hand, Tang *et al*. [40] use back-translation augmentation by translating texts from Chinese to English using the *Baidu* API to increase the number of samples in the training dataset consisting of Chinese micro-blogging texts, with English, Chinese, and Japanese texts belonging to NLPCC 2018 shared Task 1. This is part of its BERT-MSAUC model that classifies the emotions of *happiness*, *sadness*, *fear*, *anger*, and *surprise*. The results obtained by their model in the *F1-score* metric outperformed in two (*fear* and *anger*) of the five emotive classes mentioned above a BERT(M) model used as a baseline. The ranking performance for each class is shown in Table 2.

**Table 2:** *F1-score* results [40].

Emotional class	BERT(M)	BERT-MSAUC
**Joy**	77.3%	76.9%
**Sadness**	63.4%	61.4%
**Fear**	65.8%	70.6%
**Anger**	45.2%	51.3%
**Surprise**	50.1%	49.7%

To complete the reviewing of articles that use this technique, Bogoradnikova *et al*. [23] perform sentiment analysis, toxic comment detection, and toxic text part detection using the Russian language. In their research, they compare an SVM model with the Perspective API (API used for content moderation), in the first instance the performance of the SVM model is an accuracy of 61.83% after augmentation of the dataset. Then, they use EDA and back-translation to augment the dataset but do not give details of the augmentation process. After augmentation, the results with their SVM model in the sentiment analysis task improved their performance by 10% reaching 95% ranking performance in the *accuracy* metric.

### Data augmentation through generation

In this section, we will review the Generative Adversarial Networks (GANs) techniques, and the one based on vector space manipulation.

#### Generative Adversarial Networks (GANs)

GANs use generation models that create synthetic data from an existing dataset. In this sense, GANs base their operation on the use of a generator that creates sentences from an existing dataset and a discriminator, which judges whether the sentences created by the generator are real or false. When the discriminator cannot differentiate whether a sentence is real or false, we are in the presence of a sample to be added to the augmented dataset. This type of generative model commonly uses DL models for both the generator and discriminator implementation, so they rely on the training dataset to generate excellent-quality sentences [42–45].

### Augmentation by vector space manipulation

This technique, unlike sentence manipulation methods that work directly on text, vector space manipulation (embedding) works at the level of the representative vectors of the sentences in the vector space of the model used. Given this mode of operation, they are very dependent on the model to be used because the architecture of the model assumes how the sentences will be represented. Its implementation is based on neural networks and there is less research on it [17].

Based on the literature review on augmentation techniques, the overview shown in Table 3 is presented, detailing the Data Augmentation techniques, the number of articles, and their identification.

**Table 3:** Distribution of articles by augmentation method.

DA technique	Number of articles	Item Identification
**Transformation**	31	[1, 2, 4–7, 10–14, 19–22, 24–28, 30–33, 46–51]
**Paraphrase**	6	[8, 35, 40, 41, 52, 53]
**Generation**	9	[38, 39, 42–45, 54–56]
**Vector space**	3	[15, 57, 58]
**BO-TextAutoAugment**	1	[34]
**Hybrid**	2	[23, 59]
**Not specified**	1	[60]
**Comparative**	1	[17]

From the table above, it can be deduced that the most used category of augmentation techniques is sentence transformation with 57.4%, followed by the category of sentence generation with 16.6%. Thirdly, you can find the category of sentence paraphrasing with 11.1%. Concerning the language, English corresponds to the most used with 57% distantly followed by Spanish with 7.1%. Regarding the classifiers that were used in the reviewed works, the most used classification model is BERT and its variants with 23%, followed by LSTM with 15%, showing a tendency towards DL classification models.

## Method

**Fig 3:**
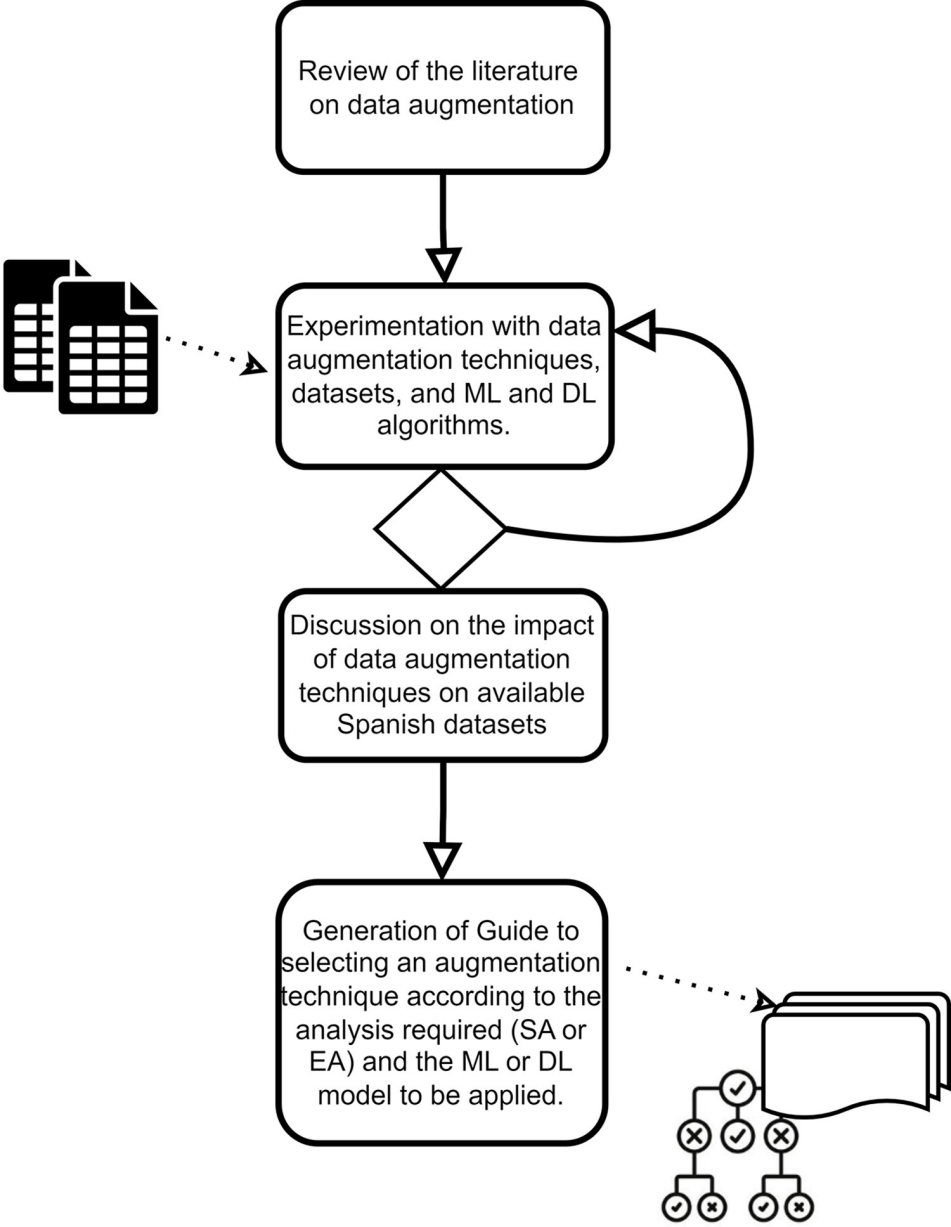
Method of work.

To confirm the hypothesis of the work, the following activities were considered for the work method (Fig 3). The following subtasks are included within the experimental phase:

1. Selection of datasets for Spanish text analysis that will be augmented.
2. Selection of augmentation techniques based on the state of the art obtained.
3. Selection of the most used classification models based on ML and DL in the state of the art obtained.
4. Application of the selected ML and DL models to original datasets, to obtain the classification performance that will be used as a baseline.
5. Application of text data augmentation techniques to the original datasets, to increase the amount of labeled data in each of them.
6. Application of selected ML and DL models to augmented datasets to obtain classification performance after augmentation.
7. Evaluation of the impact on the performance of the selected augmentation techniques on the chosen datasets using the most used metrics in the state of the art.

## Experiments

**Fig 4:**
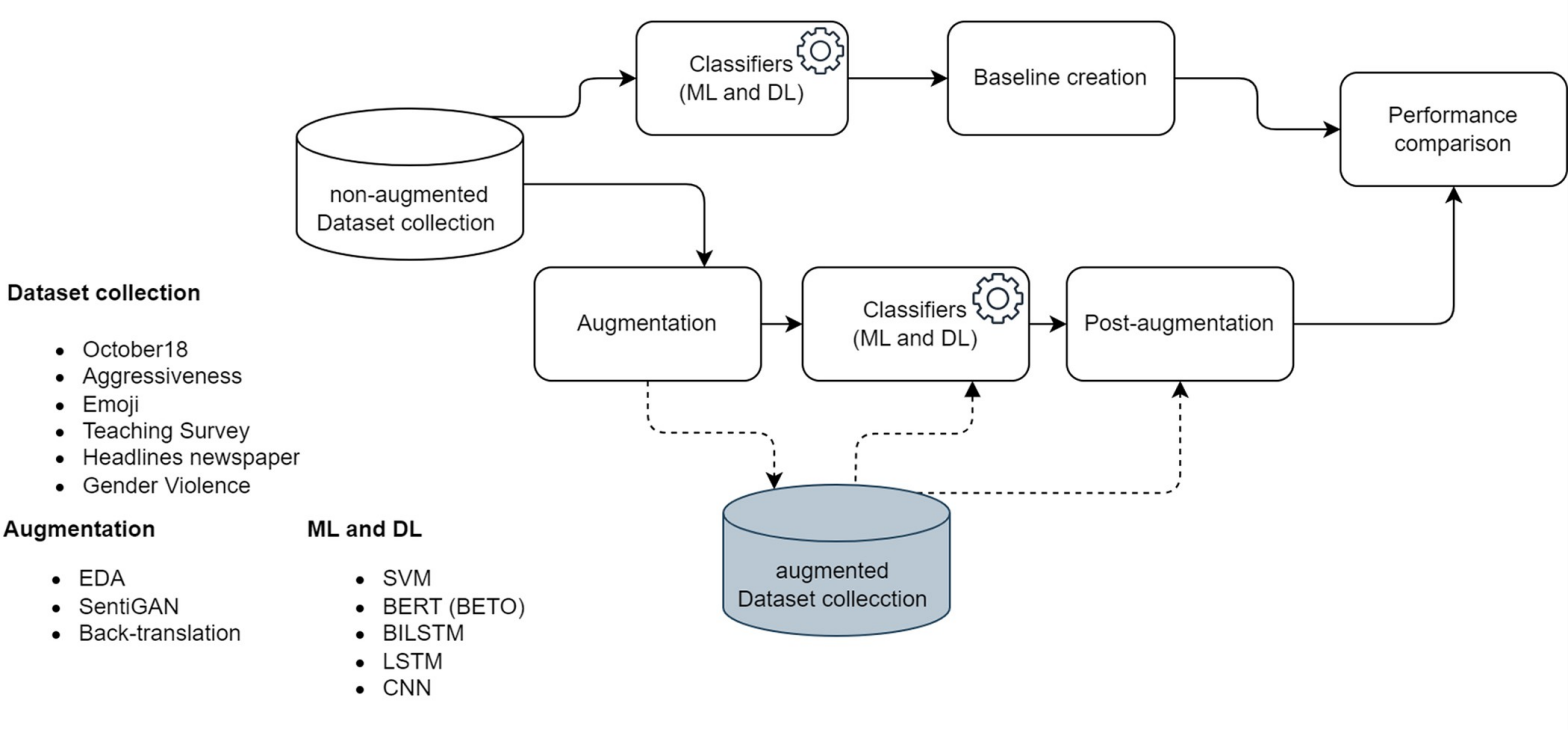
Flow of the experiments.

This section presents the details of the experiments conducted to evaluate the influence of the augmentation techniques on the datasets used. First, the classification metrics of the unaugmented corpora are calculated using different classification algorithms. These measures are called baseline and are used to compare the results obtained after classifying the augmented corpora. Fig 4 shows graphically the flow of the experimentation.

The experiments were conducted on a server featuring Debian GNU/Linux 12 (bookworm) as its operating system. It was equipped with 2 x Intel Xeon® CPU E5-2683 v4 2.10GHz processors, totaling 16 cores and 64 threads with hyperthreading enabled, along with 256 GB of RAM. Next, this section will describe six datasets made available by the SoMos group of the University of Bío-Bío that were augmented with the techniques reviewed above. In addition, the ML and DL algorithms used in the classification processes will be selected, as well as the data augmentation techniques based on the guidelines provided in the literature review.

### Selection of datasets and augmentation techniques

Among the datasets available for this study is a collection generated in earlier studies by the SoMos research group: the first, called October 18 is composed of comments collected from Twitter in the context of the social outbreak that occurred in Chile in 2019. The set is labeled with 8 categories for Emotion Analysis based on Plutchick’s taxonomy. The second set of data called Aggressiveness was used in the work of Lepe *et al*. [61] and is composed of comments from Twitter used to detect cyberbullying in Spanish. The teaching survey dataset consists of feedback provided by students in the teaching performance evaluation conducted at the University of Bio-Bío in 2018. This set, unlike the others, is labeled to classify four distinct categories for each sample (Affect, Aggressiveness, Polarity, Seriousness). That is why it was divided into four datasets, one for each category. On the other side, the Newspaper Headlines dataset was created for the work of Martínez-Araneda *et al*. [62] to analyze the bias of Chilean newspaper headlines between 2014 and 2015. At last Gender-based Violence dataset was created in the work of Calbullanca *et al*. [63, 64] and corresponds to a set of song lyrics in Spanish of different musical genres that depict violence against women. Table 4 summarizes the characteristics of the datasets.

**Table 4:** Characterization of datasets.

Description	18October	Aggressiveness [61]	Teaching Survey	Headlines News [62]	Gender Violence [63]
**Word count**	3,336	33,143	8,645	24,245	44,024
**Unique word count**	1,680	8,685	2,119	8,447	8,843
**Average of words per sentence**	17	22	5	12	44

In addition to the above, the datasets were characterized in detail according to the criteria defined in Table 5 and applied in Table 6.

**Table 5:** Criteria for detailed characterization of the dataset.

Item	Value	Description
Balanced (B)	Yes/ No	It shows if the number of samples is the same for each class.
Formal Text (TF)	Yes/ No	It shows that the area in which the text is written has restrictions on the type of words and expressions used.
Average large per sentence (LPO)	Number	Number of words in the sentence.
Size (T)	S, if Size ≤ 500; M, if Size > 500 and Size ≤ 1000; L, if Size > 1000	Categorized representation of the total number of samples per dataset.
Sample (CM)	Number	Total number of samples per dataset.
Classification task (TC)	Sentiment analysis (SA) Emotion analysis (EA)	Classification task to be solved on the dataset.
Number of classes (CC)	Number	Number of classes in a dataset.
Type of content (TC)	Comments	Type of content included in the dataset.
	Lyrics songs	
	Headlines newspaper	

**Table 6:** Detailed characterization of the dataset.

Dataset	Criteria							
	B	TF	LPO	T	CM	TC	CC	TC
**October 18**	NO	NO	17	S	195	EA	8	Comments related to October 18.
**Aggressiveness**	YES	NO	22	L	1,470	SA	2	Aggressive comments.
**Teaching Survey (Emotion)**	NO	YES	5	L	1,632	EA	7	Emotional expressions found in feedback from a teaching survey.
**Teaching Survey (Aggressiveness)**	NO	YES	5	L	1,632	SA	2	Aggressive comments are found in feedback from a teaching survey.
**Teaching Survey (Polarity)**	NO	YES	5	L	1,632	SA	3	Good and bad comments are found in feedback from a teaching survey.
**Teaching Survey (Seriousness)**	NO	YES	5	L	1,632	SA	2	Serious comments were found in feedback from the teaching survey.
**Headlines newspaper**	NO	YES	12	L	2,031	EA	9	Headlines newspapers labeled as good or unwelcome news.
**Gender-based violence**	NO	NO	44	M	1,000	SA	2	Song lyrics songs labeled as holds or not have gender-based violence against women.

Regarding the phase of selecting augmentation techniques outlined in the methodology, it is observed that the most representative technique in this category is Easy Data Augmentation (EDA) [18], known for its synonym replacement, word order change, word deletion, and word addition, was considered, except for the deletion method which, according to the EDA authors, degrades classification results.

Regarding the classification models, these were selected based on the most used in the literature review conducted. It shows that BERT is the most used classification algorithm in the reviewed articles, accounting for 23%. For our experiments, we will use BETO, a variant of BERT trained in Chilean Spanish, the same language as the data sets to be used in the experimentation. Regarding other classifiers, LSTM was used in 15% of the articles reviewed, followed by BiLSTM with 12%, and CNN with 7%. In the case of SVM, it was recommended by the SOMOS group as it has shown good classification results with the selected data sets.

#### Calculation of the baseline

The procedure used to obtain the classification performance of the datasets without augmentation that will be used as a basis of comparison for the experiments is described below. The most used algorithms in the literature review were applied to them: CNN, LSTM, BiLSTM, BERT (BETO), and SVM. 70% of the data was used for training and 30% for validation. The validation subsets were the same for all classifiers for both baseline and post-augmentation classification performance. The hyperparameters used by each of the classifiers can be seen in Table 7. These parameters remain consistent for both machine learning (ML) and deep learning (DL) models during classification, following augmentation. This ensures a fair comparison of model performance between ML and DL on both the original and augmented datasets.

**Table 7:** Hyperparameters for each classification model.

Classifier	Hyperparameter
**CNN**	Embedding_dim = 20, Conv1D (32, 3, activation = relu, padding = same), 4 MaxPooling1D (3, padding = same), Conv1D (64, 3, activation = relu, padding = same), Conv1D (64, 3, activation = relu, padding = same), 2 Conv1D (128, 3, activation = relu, padding = same), dropout = 0.5, Dense (11, activation = softmax)
**LSTM**	Embedding_dim = 16, output LSTM = 32, 1 unit LSTM, 1 Dense (19, activation = relu), 1 Dense (11, activation = softmax), loss = sparse_categorical_crossentropy, optimizer = ADAM, metrics = accuracy)
**BiLSTM**	Embedding_dim = 100, 2 units LSTM (100, dropout = 0.3), 1 Conv1D (100, 5, activation = relu), 1 Dense (16, activation = relu), 1 Dense (11, activation = softmax)
**BERT**	model_name = ’dccuchile/bert-base-spanish-wwm-cased’
	batch_size = 16, optimizer = ADAM, learning_rate = 2e-5, epochs = 10
**SVM**	param_range = [0.0001, 0.001, 0.01, 0.1, 1.0, 10.0, 100.0, 1000.0]
	param_grid = [{’svc__C’: param_range, ’svc__kernel’: [’linear’]}, {’svc__C’: param_range, ’svc__gamma’: param_range, ’svc__kernel’: [’rbf’]}]
	GridSearchCV(estimator = pipe_svm, param_grid = param_grid, scoring = ’accuracy’, cv = 10, n_jobs = -1)

Once the algorithms were applied to the datasets without augmentation, the results shown in Table 8 were obtained. In it, you can see the results with the accuracy and *F1-score* metrics for each dataset.

**Table 8:** Results for the baseline.

Dataset	Classifier									
	SVM		CNN		LSTM		BiLSTM		BERT	
	ACC	F1	ACC	F1	ACC	F1	ACC	F1	ACC	F1
**October 18**	56%	10%	56%	40%	56%	40%	56%	40%	**63%**	**70%**
**Aggressiveness**	90%	90%	82%	82%	84%	83%	58%	43%	**90%**	**91%**
**Teaching Survey (Emotion)**	57%	53%	52%	53%	**59%**	54%	33%	17%	56%	**55%**
**Teaching Survey (Aggressiveness)**	95%	92%	95%	92%	95%	92%	95%	92%	**96%**	**94%**
**Teaching Survey (Polarity)**	71%	69%	75%	75%	72%	73%	56%	40%	**80%**	**79%**
**Teaching Survey (Seriousness)**	**79%**	70%	76%	76%	**79%**	70%	**79%**	70%	77%	**73%**
**Headlines newspapers (Emotion)**	39%	**43%**	26%	23%	31%	22%	27%	11%	**44%**	**43%**
**Gender-based Violence**	**86%**	**86%**	76%	66%	83%	81%	76%	66%	83%	83%

From the results presented, the classification model that obtains the best results in either *accuracy* or *F1-score* is BERT (BETO). However, in the gender-based violence dataset, the SVM model is the one that obtains the best results in both metrics with a difference of 3% concerning its closest follower (BERT).

#### The procedure with transformation techniques

To augment the selected datasets, the training subset is augmented in two dimensions. The first is the percentage of modification over a sentence, while the second is the amount of augmentation made over the dataset. Table 9 shows the distribution, percentage, and number of augmentations applied.

**Table 9:** EDA augmentation parameters.

Modification percentage	Number of augmentations
5	1,2,3,4,5,6,7,8,9
10	1,2,3,4,5,6,7,8,9
20	1,2,3,4,5,6,7,8,9
30	1,2,3,4,5,6,7,8,9

The second experiment corresponds to the selection of the best percentage of modification after increasing with EDA and classifying, and then increasing the number of samples of the minority classes in the datasets using that percentage to perform dataset balancing.

#### The procedure with generative techniques

Augmentation experiments with generative techniques were performed using generative adversarial networks (GANs) using the SentiGAN model proposed by Wang and Wan [65]. To augment this model, the classes of each dataset were separated into separate files and then augmented one by one. Augmentation with SentiGAN was done because although the model claims to be prepared to generate augmentation for multiple classes present in a dataset, in practice it can only generate for a single class. Table 10 shows the configuration parameters employed for augmentation using SentiGAN.

**Table 10:** SentiGAN configuration parameters.

**Generator**	
**EMB_DIM**	200
**HIDDEN_DIM**	200
**MAX_SEQ_LENGTH**	Average words per sentence in the dataset
**BATCH_SIZE**	Number of sentences per class
**Discriminator**	
**DISM_EMBEDDING_DIM**	64
**DIS_FILTER_SIZES**	[1, 2, 3, 4, 5, 6, 7, 8, 9, 10, 15]
**DIS_NUM_FILTERS**	[100, 200, 200, 200, 200, 100, 100, 100, 100, 100]
**DIS_DROPOUT_KEEP_PROB**	0.75
**DIS_L2_REG_LAMBDA**	0.2
**DIS_BATCH_SIZE**	Number of Prayers per Class
**TOTAL_BATCH (adversarial process)**	100

#### The procedure with paraphrasing techniques

Experiments to augment the datasets using paraphrasing techniques were conducted using Google’s translation service. To begin with, the datasets in xlsx format were uploaded to the site, and then the translation was downloaded in three languages (English, German, and French). The next step was to upload the translated documents and translate them back into Spanish. Thus, slightly modified sentences were added to the training dataset for the classification models used. The datasets were incrementally augmented with the results obtained from the translation, according to Table 11.

**Table 11:** Augmentation levels with back-translation.

Augmentations	Languages
1	Base (Spanish) + English
2	Base (Spanish) + English + German
3	Base (Spanish) + English + German + French

## Results

From the results of the experiments conducted on the sets of texts in Spanish described in Table 6, the following guidelines were generated for the selection of augmentation techniques in the form of a decision tree from the point of view of the classification task addressed by the dataset. It should be noted that this tree is presented in a descriptive and non-predictive manner given the volume of data and instances for each class of the result dataset.

**Fig 5:**
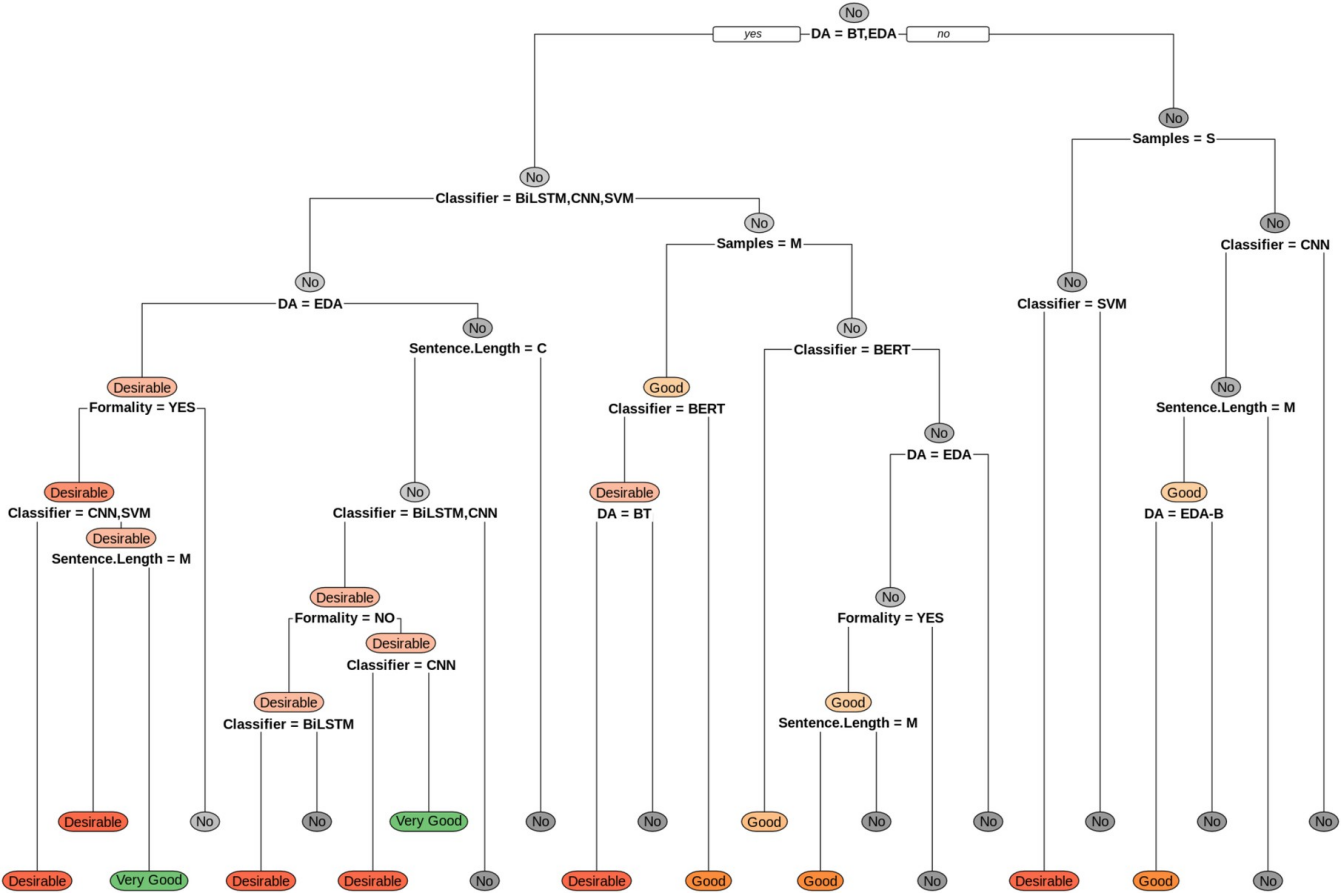
Guide to selecting data augmentation techniques for emotion analysis.

**Fig 6:**
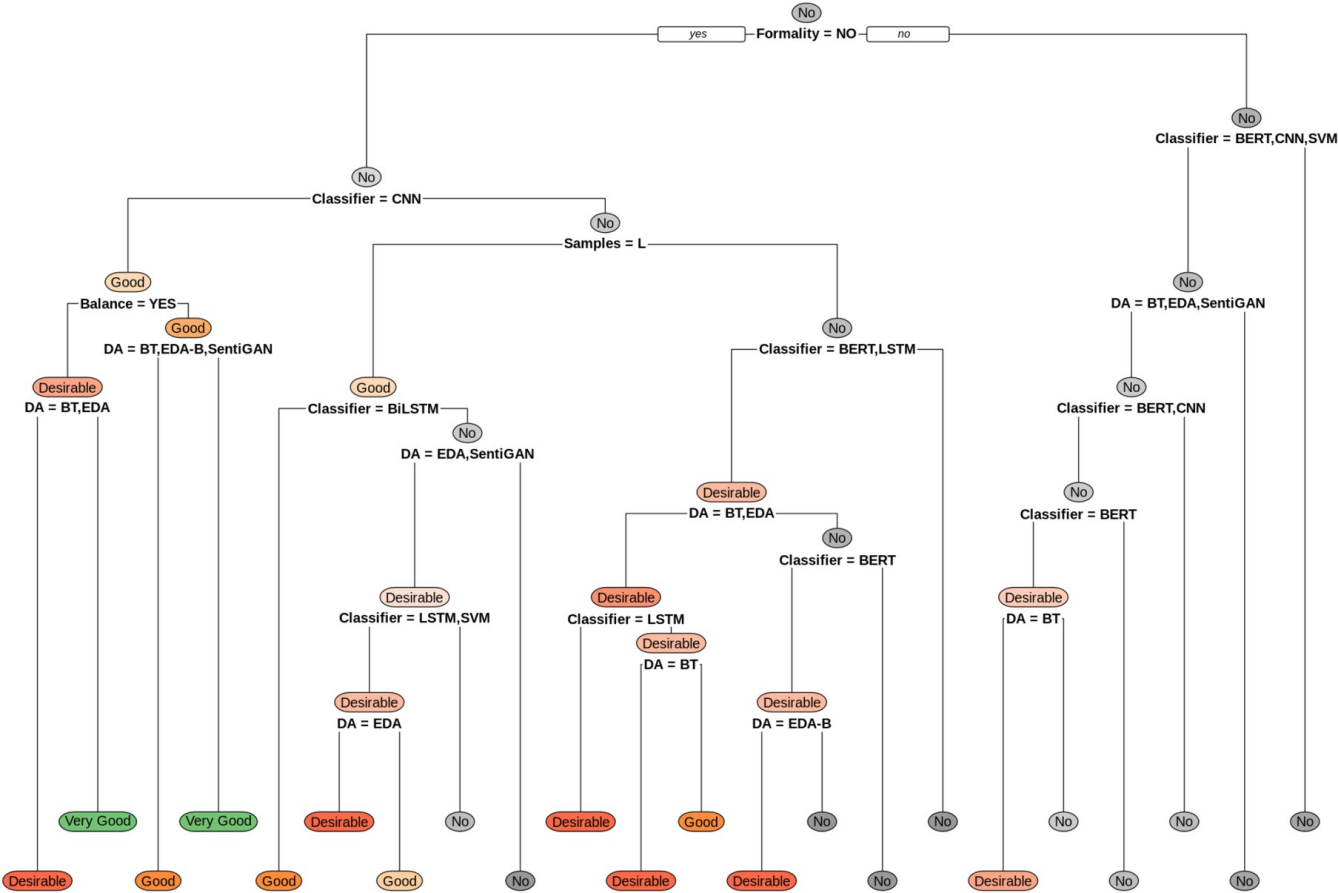
Guide to selecting data augmentation techniques for sentiment analysis.

The first decision tree (Fig 5) is the selection of the augmentation technique based on the impact it has on the classification performance for the EA classification task. On the other hand, the second decision tree (Fig 6) presents the best paths in selecting an augmentation technique according to the impact on performance for the baseline for the datasets for the SA classification task.

Table 12 shows the results of augmentation with the selected techniques and makes a comparison between them and the baseline. The DA approaches included are:

- **EDA**: Transformation augmentation technique.
- **EDA-B**: Balancing classes using EDA.
- **SentiGAN**: Generation augmentation technique.
- **BT**: Augmentation technique by back-translation paraphrasing.

**Table 12:** Performances of text augmentation approaches.

Dataset	DA approach	Classifier				
		SVM	CNN	LSTM	BiLSTM	BETO^1^
**October 18**	**Base**	56%	56%	56%	56%	63%
	**EDA**	**54%**	58%	56%	56%	67%
	**EDA-B**	N/A	N/A	N/A	N/A	N/A
	**BT**	**54%**	56%	56%	56%	**73%**
	**SentiGAN**	58%	44%	42%	17%	3%
**Aggressiveness**	**Base**	90%	82%	84%	58%	90%
	**EDA**	91%	83%	85%	67%	89%
	**EDA-B**	N/A	N/A	N/A	N/A	N/A
	**BT**	89%	83%	**82%**	64%	90%
	**SentiGAN**	99%	**100%**	99%	64%	94%
**Teaching Survey (Emotion)**	**Base**	57%	52%	59%	33%	56%
	**EDA**	58%	54%	57%	**46%**	61%
	**EDA-B**	N/A	N/A	N/A	N/A	N/A
	**BT**	56%	55%	58%	**46%**	56%
	**SentiGAN**	43%	43%	25%	13%	4%
**Teaching Survey (Aggressiveness)**	**Base**	95%	95%	95%	95%	96%
	**EDA**	95%	95%	95%	95%	96%
	**EDA-B**	93%	91%	90%	5%	**4%**
	**BT**	94%	92%	94%	95%	94%
	**SentiGAN**	90%	92%	95%	95%	96%
**Teaching Survey (Polarity)**	**Base**	71%	75%	72%	56%	80%
	**EDA**	75%	76%	**77%**	56%	82%
	**EDA-B**	75%	69%	71%	56%	78%
	**BT**	75%	74%	76%	56%	81%
	**SentiGAN**	73%	71%	66%	**31%**	72%
**Teaching Survey (Seriousness)**	**Base**	79%	76%	79%	79%	77%
	**EDA**	79%	76%	79%	79%	**81%**
	**EDA-B**	76%	75%	75%	21%	**20%**
	**BT**	78%	77%	79%	79%	80%
	**SentiGAN**	79%	79%	79%	79%	80%
**Headlines newspapers**	**Base**	39%	26%	31%	27%	44%
	**EDA**	41%	27%	36%	30%	43%
	**EDA-B**	38%	**33%**	25%	**8%**	25%
	**BT**	39%	26%	31%	27%	48%
	**SentiGAN**	34%	26%	29%	22%	42%
**Gender-based Violence**	**Base**	86%	76%	83%	76%	83%
	**EDA**	86%	**87%**	85%	76%	87%
	**EDA-B**	86%	85%	**73%**	76%	84%
	**BT**	86%	86%	84%	76%	86%
	**SentiGAN**	85%	85%	81%	50%	78%

^1^Based on BERT model

The findings show that the Convolutional Neural Network (CNN) classifier achieved the most significant improvement, with an 18% increase using the Generative Adversarial Networks for Sentiment Text (SentiGan) on the Aggressiveness dataset. Additionally, the same classifier model showed an 11% improvement using the Easy Data Augmentation (EDA) on the Gender-Based Violence dataset. The performance of the Bidirectional Encoder Representations from Transformers (BETO) also improved by 10% on the back-translation augmented version of the October 18 dataset, and by 4% on the EDA augmented version of the Teaching survey dataset.

Another important result of this work is related to the guidelines for the selection of techniques. These consist of a series of rules obtained from the analysis of the results of this work, which provide guidance when applying data augmentation techniques. They were classified into four levels according to the degree of improvement in the results obtained from:

The characteristics of the data set (corpus) used: size of the set, sentence size, formality of language.

- The type of augmentation technique to be used.
- The classification algorithm to be applied.
- The approach to analysis, i.e., Sentiment Analysis (classification into two classes) or Emotion Analysis (classification of more than two classes).

For example, the rule presented as ***"Good WHEN DA IS BT OR EDA & Classification IS LSTM & Samples IS M"*** describes that for a medium (M) sized corpus, regardless of the formality of the text or the average length of the sentences, if the LSTM classification algorithm is used, it is suggested that data augmentation using the EDA or BT techniques be used to obtain an increase of between 3% and 5% in classification performance compared to not using data augmentation. This is in the case of Emotion Analysis (EA).

In cases where the data augmentation obtains good results, rules such as those described in Table 13 are obtained.

**Table 13:** Examples of EA selection rules (positive results).

**Emotion analysis**	Good WHEN	DA IS	BT or EDA	&	Classification IS	BERT	&	Sample IS	L or S			
	Good WHEN	DA IS	BT or EDA	&	Classification IS	LSTM	&	Sample IS	M			
	Good WHEN	DA IS	EDA	&	Classification IS	LSTM	&	Sample IS	L	&	SentenceSize IS	M
	Good WHEN	DA IS	EDA-B	&	Classification IS	CNN	&	Sample IS	L or M	&	SentenceSize IS	M
	Desirable WHEN	DA IS	BT	&	Classification IS	BERT	&	Sample IS	M			
	Desirable WHEN	DA IS	EDA	&	Classification IS	CNN or SVM	&	Sample IS	L			
	Desirable WHEN	DA IS	EDA	&	Classification IS	BiLSTM	&	Sample IS	L	&	SentenceSize IS	M
	Desirable WHEN	DA IS	BT	&	Classification IS	BiLSTM	&	Sample IS	M	&	SentenceSize IS	C
	Desirable WHEN	DA IS	BT	&	Classification IS	CNN	&	Sample IS	L	&	SentenceSize IS	C
	Desirable WHEN	DA IS	EDA-B or SentiGAN	&	Classification IS	SVM	&	Sample IS	S			
	Very Good WHEN	DA IS	EDA	&	Classification IS	BiLSTM	&	Sample IS	L	&	SentenceSize IS	C
	Very Good WHEN	DA IS	BT	&	Classification IS	BiLSTM	&	Sample IS	L	&	SentenceSize IS	C

In cases where the data augmentation obtains poor results, rules such as those described in Table 14 are obtained.

**Table 14:** Examples of EA selection rules (negative results).

**Emotion analysis**	No WHEN	DA IS	EDA	&	Classification IS	BiLSTM or CNN or SVM	&	Sample IS	M or S			
	No WHEN	DA IS	EDA	&	Classification IS	BERT	&	Sample IS	M			
	No WHEN	DA IS	EDA	&	Classification IS	LSTM	&	Sample IS	L	&	SentenceSize IS	C
	No WHEN	DA IS	EDA	&	Classification IS	LSTM	&	Sample IS	S			
	No WHEN	DA IS	BT	&	Classification IS	LSTM	&	Sample IS	L or S			
	No WHEN	DA IS	BT	&	Classification IS	SVM	&	Sample IS	L	&	SentenceSize IS	C
	No WHEN	DA IS	BT	&	Classification IS	CNN or SVM	&	Sample IS	M	&	SentenceSize IS	C
	No WHEN	DA IS	BT	&	Classification IS	BiLSTM or CNN or SVM	&	SentenceLarge IS	M			
	No WHEN	DA IS	EDA-B or SentiGAN	&	Classification IS	BiLSTM or CNN or LSTM	&	Sample IS	S			
	No WHEN	DA IS	SentiGAN	&	Classification IS	CNN	&	IS Samples	L or M	&	SentenceSize IS	M
	No WHEN	DA IS	EDA-B or SentiGAN	&	Classification IS	CNN	&	IS Samples	L or M	&	SentenceSize IS	C
	No WHEN	DA IS	EDA-B or SentiGAN	&	Classification IS	BERT or BiLSTM or LSTM or SVM	&	IS Samples	L or M			

In cases where the data augmentation obtains good results, rules such as those described in Table 15 are obtained.

**Table 15:** Examples of SA selection rules (positive results).

**Sentiment Analysis**	good WHEN	SentenceSize IS	L	&	Classification IS	CNN	&	DA IS	BT or EDA-B or SentiGAN
	good WHEN	SentenceSize IS	M	&	Classification IS	BiLSTM			
	good WHEN	SentenceSize IS	M	&	Classification IS	SVM	&	DA IS	SentiGAN
	good WHEN	SentenceSize IS	L	&	Classification IS	BERT	&	DA IS	EDA
	Desirable WHEN	SentenceSize IS	C	&	Classification IS	BERT	&	DA IS	BT
	Desirable WHEN	SentenceSize IS	M	&	Classification IS	CNN	&	DA IS	BT or EDA
	Desirable WHEN	SentenceSize IS	M	&	Classification IS	LSTM or SVM	&	DA IS	EDA
	Desirable WHEN	SentenceSize IS	L	&	Classification IS	BERT	&	DA IS	BT
	Desirable WHEN	SentenceSize IS	L	&	Classification IS	LSTM	&	DA IS	BT or EDA
	Desirable WHEN	SentenceSize IS	L	&	Classification IS	BERT	&	DA IS	EDA-B
	Very Good WHEN	SentenceSize IS	L	&	Classification IS	CNN	&	DA IS	EDA
	Very Good WHEN	SentenceSize IS	M	&	Classification IS	CNN	&	DA IS	SentiGAN
	Very Good WHEN	SentenceSize IS	M	&	Classification IS	LSTM	&	DA IS	SentiGAN

In cases where the data augmentation obtains poor results, rules such as those described in Table 16 are obtained.

**Table 16:** Examples of SA selection rules (negative results).

**Sentiment Analysis**	No WHEN	SentenceSize IS	C	&	Classification IS	BERT	&	DA IS	EDA or SentiGAN
	No WHEN	SentenceSize IS	M	&	Classification IS	BERT	&	DA IS	EDA or SentiGAN
	No WHEN	SentenceSize IS	C	&	Classification IS	CNN	&	DA IS	BT or EDA or SentiGAN
	No WHEN	SentenceSize IS	C	&	Classification IS	SVM	&	DA IS	BT or EDA or SentiGAN
	No WHEN	SentenceSize IS	C	&	Classification IS	BERT or CNN or SVM	&	DA IS	EDA-B
	No WHEN	SentenceSize IS	C	&	Classification IS	BiLSTM or LSTM			
	No WHEN	SentenceSize IS	M	&	Classification IS	BERT or LSTM or SVM	&	DA IS	BT
	No WHEN	SentenceSize IS	L	&	Classification IS	BERT	&	DA IS	SentiGAN
	No WHEN	SentenceSize IS	L	&	Classification IS	LSTM	&	DA IS	EDA-B or SentiGAN
	No WHEN	SentenceSize IS	L	&	Classification IS	BiLSTM or SVM			

## Discussion

When analyzing the impact of the increase on ranking performance, the following can be seen:

- One of the determining factors in the impact that augmented datasets have on classification performance is how different the samples created by augmentation are. In all the experiments performed, diversity was a determining factor when increasing the performance of the classifiers over the number of artificial samples created with augmentation.
- Another factor that affects the quality of the samples artificially created with augmentation techniques is the average length of the sentence. When transformation or generation techniques are used, if a dataset has sentences with an average of fewer words than 15 words, there is a minor or negative impact on classification performance.
- The technique of augmentation by paraphrasing, and back-translation, has a positive impact on most datasets by adding a sentence like the existing one but syntactically distinct enough that it is not the same but that converses with the semantics, thus preventing classifiers from being trained on datasets with similar samples, which leads to overfitting.

Another relevant topic when analyzing the impact of augmentation techniques on ranking performance is the challenges faced when applying these techniques. Among the challenges met in applying augmentation techniques are:

- The augmentation techniques used and reviewed are not trained in the Spanish language. Therefore, it was necessary to change them in a way that could augment the datasets described in the Experiments section. In the case of EDA, this modification was the change of the lexical dictionary used by the technique and the change of the stop words in English for their Spanish version. On the other hand, SentiGAN was trained with different datasets in English with a larger number of samples and with an average number of words per sentence higher than the datasets used in this work, therefore, it was necessary to retrain the model with the datasets in Spanish.
- Another challenge met with SentiGAN was that the documentation of the technique that goes with the code in the GitHub repository is https://github.com/Nrgeup/SentiGAN scarce and does not conform to what is expressed in the work of Wang and Wan [65] which shows that SentiGAN is ready for the generation of sentences for multiple classes. In practice, the code provided in the repository is prepared to generate samples for only one class. This harmed the time needed for the execution of the experiments conducted in this study.
- An additional challenge was that the generative augmentation techniques analyzed in the state of the art are written in versions of Python and dependencies that are no longer in force. For example, SentiGAN was written in Python 2.7 and uses TensorFlow 1.4. These requirements made it necessary to create virtual environments that could execute the technique and that had sufficient computing power for its execution.

## Conclusions

This work contributes to the advancement of natural language processing through a framework that guides the selection of extension techniques, which artificially augment datasets in Spanish, specifically for classification tasks. To achieve this purpose, an exhaustive analysis of the current state of text augmentation was conducted through an extensive review of the literature. This analysis allowed us to understand that text enlargement involves a series of methods used to artificially augment labeled datasets, and the techniques were classified according to the manipulation performed on the data. Experiments with transformation, generation, and paraphrasing techniques show the following:

1. Depending on the augmentation technique and the classifier used, it is possible to improve the classification performance.
2. For datasets related to emotion analysis, the back-translation paraphrasing technique is one of the best options, regardless of the characteristics of the dataset.
3. In data augmentation with EDA, the percentage of word modification in a sentence is a crucial parameter for adding diversity to the dataset, but EDA does not guarantee the preservation of semantics, suggesting exploring other EDA-based amplification techniques.
4. Balancing datasets with EDA can significantly decrease the performance of classifiers due to sentence quality and poor original performance.
5. Generative techniques show good results on sizable and balanced datasets, especially for the SA task when using the CNN classifier.

The proposed selection guidelines are considered a starting point for the choice of Spanish text augmentation techniques, subject to improvements as more experiments are conducted to extend them to a greater number of features present in the Spanish datasets.

In conclusion, the application of augmentation techniques improves the classification performance in various DL and ML models for Spanish data sets, despite their smaller size compared to English data sets. The results and code are available in the GitLab repository https://gitlab.com/rgutierrezb/dataaugmentation.

## Future works

Given the results obtained in the balancing of classes with EDA, in the future, it is desirable to explore the effects of balancing by increasing the sets of texts that address the task of analyzing emotions in Spanish (EA) to improve the results obtained in this work.

The augmentation techniques used in this work correspond to the most representative of the categories of transformation, generation, and paraphrasing belonging to the taxonomy [17], so future work can be considered experimenting with the remaining techniques of this taxonomy, especially with the use of Large Language models (LLMs) such as GPT (OpenAI), Llama (Meta), and Claude (Anthropic), among others.

In a more practical sense, future work contemplates the implementation of a web application based on natural language processing in the Spanish language.
